# The Future Is Now

**DOI:** 10.1289/ehp.1205032

**Published:** 2012-03-01

**Authors:** Hugh A. Tilson, Jane C. Schroeder, Susan M. Booker

**Affiliations:** Editor-in-Chief, *EHP*, E-mail: tilsonha@niehs.nih.gov; Science Editor, *EHP*; News Editor, *EHP*

In 1972 David P. Rall, director of the National Institute of Environmental Health Sciences (NIEHS), determined there was a need for a journal dedicated to research on human health and the environment, and *Environmental Health Perspectives* (*EHP*) was born. The journal published monographs on topics related to environmental human health and research papers based on conference proceedings for more than 20 years before adopting its current format in 1993 to include news, correspondence, editorials, reviews, commentaries, and research articles. In 2004 *EHP* became one of the first open-access scientific journals, with all news and research content available for free on the journal’s website (Olden and Goehl 2004). In keeping with this progressive tradition, we are pleased to announce the next phase of *EHP’*s evolution: Starting with the January 2013 issue, *EHP* will become an exclusively online journal. This decision has the full support of management at the NIEHS, the *EHP* Advisory Board, and the *EHP* Board of Associate Editors.

There are several reasons for going to an online-only format at this time. First and foremost, going paperless will allow us to focus on optimizing electronic delivery of *EHP*, which is how the vast majority of our readers access the journal now. In 2011 the *EHP* website had more than 1.2 million unique visitors and 2.7 million page views, and there were another 1.4 million visits to *EHP* content via PubMed Central. In contrast, there were approximately 600 paid subscribers to the print version of the journal and a total print circulation of 2,500 copies. Clearly, *EHP* already reaches a much larger audience online than in print, and our time, effort, and resources should be directed accordingly.

Going paperless also will increase the flexibility of the journal and allow us to concentrate on enhancing the *EHP* website and expanding options for electronic delivery. *EHP* is currently available via a free iPhone application. An Android application is under development, and we are planning tablet and mobile site applications as well. A fully digital format will increase opportunities to include video and audio material in both news and research articles. In addition, we are planning a more dynamic format for the *EHP* News section, which will help attract additional readers and increase the breadth of our audience.

There are also economic advantages to going paperless. Libraries and research institutions are cancelling print subscriptions to journals, including *EHP*, because of storage space and costs, and our number of individual print subscribers is decreasing as readers become increasingly comfortable with the free online version of the journal. Consequently, our revenue from print subscriptions is decreasing, while costs associated with printing and distributing the journal continue to increase. Of course, there will still be significant costs associated with producing a paperless version of the journal. This is especially true since *EHP* is committed to maintaining its high-quality layout and graphics, continuing to publish superior news content, and upgrading its website capabilities. Therefore, we will continue to receive support from the NIEHS and fees from authors to help pay for the cost of processing, reviewing, editing, and publishing *EHP* online.

Finally, although any publishing enterprise (including online publishing) involves some degree of environmental impact, moving to an online-only format will substantially reduce *EHP*’s use of paper and ink, eliminate the need to transport and distribute print copies of the journal, and reduce the journal’s contribution to the waste stream. Going paperless is also consistent with the goals of Executive Order 13589 (Obama 2011), which was signed 9 November 2011 by President Barack Obama to promote efficient spending in the Federal government. By going paperless, *EHP* will help reduce expenditures related to continued or new printing of publications and act in accordance with the commitment to sustainability of the National Institutes of Health.

There are many advantages to moving to a paperless format, but above all, it will allow *EHP* to continue to broaden its dissemination and increase the impact of its environmental health information without compromising its high standards of peer review and rigorous editorial oversight. We believe scientific publishing is headed toward a future in which paperless journals are the industry standard. For *EHP*, the future is now.
